# Effects of Different Doses, Forms, and Frequencies of Zinc Supplementation on Biomarkers of Iron and Zinc Status among Young Children in Dhaka, Bangladesh

**DOI:** 10.3390/nu14245334

**Published:** 2022-12-15

**Authors:** M. Munirul Islam, Robert E. Black, Nancy F. Krebs, Jamie Westcott, Julie M. Long, Kazi M. Islam, Janet M. Peerson, Rahvia Alam Sthity, Afsana Mim Khandaker, Mehedi Hasan, Shams El Arifeen, Tahmeed Ahmed, Janet C. King, Christine M. McDonald

**Affiliations:** 1 Nutrition and Clinical Services Division, International Centre for Diarrhoeal Disease Research, Bangladesh (icddr,b), Dhaka 1212, Bangladesh; 2 International Zinc Nutrition Consultative Group, University of California, San Francisco, CA 94609, USA; 3 Bloomberg School of Public Health, Institute for International Programs, Johns Hopkins University, Baltimore, MD 21218, USA; 4 Department of Pediatrics, Denver Anschutz Medical Campus, University of Colorado, Aurora, CO 80045, USA; 5 Independent Consultant, Davis, CA 95616, USA; 6 Division of Nutritional Sciences and Toxicology, University of California, Berkeley, CA 94720, USA; 7 Division of Gastroenterology, Hepatology and Nutrition, Department of Pediatrics, School of Medicine, University of California, San Francisco, CA 94609, USA

**Keywords:** zinc, micronutrients, biomarker, Bangladesh

## Abstract

Young children in resource-constrained settings are susceptible to zinc deficiency and its deleterious health effects. The objective of this secondary analysis was to evaluate the effects of the following six interventions on biomarkers of iron and zinc status among a subgroup of young children in Dhaka, Bangladesh, who participated in the Zinc in Powders Trial (ZiPT): (1) standard micronutrient powders (MNPs) containing 4.1 mg zinc and 10 mg iron, daily; (2) high-zinc (10 mg) and low-iron (6 mg) (HiZn LoFe) MNP, daily; (3) HiZn (10 mg) and LoFe (6 mg)/HiZn (10 mg) and no-iron MNPs on alternating days; (4) dispersible zinc tablet (10 mg), daily; (5) dispersible zinc tablet (10 mg), daily for 2 weeks at enrollment and at 12 weeks; (6) placebo powder, daily. At the end of the 24 week intervention period, children in the daily dispersible zinc tablet group exhibited a mean serum zinc concentration (SZC) of 92.5 μg/dL, which was significantly higher than all other groups except the HiZn LoFe MNP alternating group (81.3 μg/dL). MNPs containing 10 mg and 6 mg of iron had a similar impact on biomarkers of iron status, with no evidence of an adverse interaction with zinc.

## 1. Introduction

Zinc deficiency remains one of the most common micronutrient deficiencies globally. Although nationally representative biochemical data on zinc status remain limited, it is estimated that 17% of the global population is at risk of inadequate zinc intake [1]. Among the 25 countries for which national plasma/serum zinc data are available, 23 had a zinc deficiency prevalence of >20% for at least one physiological group [2]. Young children in resource-constrained settings are especially susceptible to zinc deficiency given the elevated nutrient requirements to support growth and development and the array of socioeconomic and cultural factors that can limit access to and intake of foods that are rich in bioavailable zinc.

Zinc deficiency in young children can lead to impaired growth, increased incidence of diarrhea and other infectious morbidity, and an elevated risk of mortality [3,4]. In 2011, approximately 116,000 child deaths were attributable to zinc deficiency worldwide [5]. A variety of strategies exist for the improvement of zinc nutrition and related health outcomes in nutritionally vulnerable groups. In addition to approaches that promote optimal infant and young child feeding practices and the consumption of local nutrient-dense foods, point-of-use fortification of complementary foods with micronutrient powders (MNPs) and daily supplementation with dispersible zinc tablets may help to narrow gaps in dietary zinc intake and improve zinc status among young children. Systematic reviews of trials of preventive zinc supplementation among children older than 6 months of age have reported reductions in the incidence of diarrhea and small increases in height [3,4]. However, the results of more recent research have been mixed, and the World Health Organization (WHO) has not developed recommendations for the use of preventive zinc supplements in young children. In contrast, WHO guidance regarding the use of MNPs has been in place since 2011 and MNPs are used in many countries as a means of improving the micronutrient content in the diets of young children [6,7,8]. Although MNPs were originally designed to improve iron status, the standard MNP formulation currently includes 15 micronutrients, including zinc [9,10]. While there is robust and consistent evidence to support the use of MNPs as a strategy to reduce iron deficiency, less evidence is available regarding the effects of MNPs on serum zinc concentrations (SZC) [11,12]. A 2013 systematic review revealed that MNPs were associated with a significant increase in diarrhea [13], and some research has shown that the iron content of MNPs may have detrimental effects on the gut microbiome [14]. MNPs also appear to have limited benefits for growth and morbidity [11,12].

We previously reported the results of a randomized, partially double-blind, controlled, community-based efficacy trial that evaluated the effects of different doses, durations, and frequencies of zinc supplementation on the incidence of diarrhea and the change in linear growth among young children in Dhaka, Bangladesh [15,16]. This article reports the results of a secondary analysis, which explored the effects of the different study regimens on changes in biomarkers of iron and zinc status among a randomly selected subgroup of participants over the 24 week intervention period.

## 2. Materials and Methods

### 2.1. Study Design

This was a sub-study of a randomized, partially double-blind, placebo-controlled, community-based efficacy trial that evaluated the effects of different doses, forms, and frequencies on the incidence of diarrhea and the change in linear growth among young children in the Mirpur area of Dhaka, Bangladesh [15,16]. The primary aim of the sub-study was to compare the change in mean concentrations of biomarkers of iron and zinc status and the corresponding change in the prevalence of iron and zinc deficiency over the 24 week intervention period among a subgroup of trial participants randomly selected to be part of the biochemical subgroup.

### 2.2. Participants

A comprehensive description of trial procedures and participants has been published previously [15,16]. To summarize, children were eligible to participate in the parent trial if they were 9–11 months of age at enrollment. The following exclusion criteria were applied: (1) severe acute malnutrition, defined as weight-for-length *Z*-score (WLZ) < −3 according to the 2006 WHO Growth Standards, mid-upper arm circumference (MUAC) < 115 mm, or the presence of bipedal edema; (2) hemoglobin concentration < 8.0 g/dL; (3) congenital anomalies or any other conditions that could interfere with feeding; (4) chromosomal anomalies and other organic problems (e.g., jaundice, tuberculosis, etc.). Children who met the eligibility criteria and whose caregivers provided informed consent were randomly assigned to one of the six intervention groups described below. At the time of enrollment and randomization, approximately 58 children per intervention group were randomly selected for inclusion in the biochemical subgroup.

### 2.3. Interventions

The six intervention groups were as follows: (1) standard 15 component MNP containing 4.1 mg zinc and 10 mg iron, consumed daily; (2) high-zinc (10 mg) and low-iron (6 mg) (HiZn LoFe) MNPs, consumed daily; (3) HiZn LoFe/high-zinc (10 mg) and no-iron MNPs, consumed on alternating days; (4) dispersible tablets with 10 mg zinc, consumed daily; (5) intermittent zinc (dispersible tablets with 10 mg zinc consumed daily for 2 weeks at enrollment and at 12 weeks); and (6) placebo powder, consumed daily. The duration of the intervention was 24 weeks. More details regarding the study interventions are provided in Table S1. All child participants who suffered from diarrhea at any point throughout the trial received therapeutic zinc supplementation and oral rehydration solution (ORS) in accordance with Bangladesh’s national policy and WHO guidelines, and other supplements were suspended until the completion of treatment [17,18].

### 2.4. Study Procedures

To screen for eligibility criteria, trained study anthropometrists performed weight and length measurements of all potential participants, and length-for-age *Z*-scores (LAZ), WLZ, and weight-for-age *Z*-scores (WAZ) were calculated following the 2006 WHO Growth Standards [19]. Hemoglobin concentrations were also measured from a fingerprick blood sample using a Hemocue 301+ instrument (Hemocue, Ångelholm, Sweden) to screen for severe anemia (hemoglobin < 8.0 g/dL). At the time of enrollment, study personnel recorded background characteristics and conducted a two-week morbidity recall from all study participants.

At enrollment and upon completion of the 24 week study period, a trained phlebotomist collected 5 mL of venous whole blood from the antecubital vein of participants in the biochemistry subgroup. Blood samples were drawn in the morning hours when the participants were in a fasted state and were collected into certified trace element-free tubes (Sarstedt, Numbrecht, Germany) using aseptic techniques and IZiNCG recommendations for the avoidance of contamination [20]. Collected blood samples were immediately refrigerated and centrifuged at 3000 rpm for 10 min between 30 and 60 min of the time of collection for separation of serum. Serum samples were then aliquoted into two tubes: (1) a trace element-free cryovial for analysis of SZC, and (2) a Sarstedt cryovial for analysis of ferritin, soluble transferrin receptor (sTfR), retinol-binding protein (RBP), alpha-1-glycoproetin (AGP), and C-reactive protein (CRP). These aliquots were stored at −20 °C and later shipped to the Pediatric Nutrition Laboratory, University of Colorado, and the VitMin Laboratory (Willstaet, Germany) for laboratory analysis. In addition, capillary blood samples were collected and hemoglobin concentrations were measured using a Hemocue 301+ (Hemocue; Ångelholm, Sweden) among all study participants after 12 and 24 weeks of intervention.

### 2.5. Laboratory Analysis Methods

SZC was measured in the Pediatric Nutrition Lab at the University of Colorado using a 30 µL sample on an ICP-Mass Spectrometry device (ICP-MS, Agilent 7700x, Santa Clara, CA, USA), as previously described. Ferritin, sTfR, RBP, AGP, and CRP were analyzed in the VitMin Laboratory in Germany using sandwich ELISA [21].

### 2.6. Statistical Analyses

The sample size calculation for the biochemistry subgroup was based on an expected standard deviation of SZC of 19.6 µg/dL, 80% power, alpha of 0.05, and a potential loss of 15% of samples due to attrition, sampling error, or insufficient blood collection. With these parameters, 58 children per group were necessary to detect a difference in SZC of 10.9 µg/dL between any two groups.

Data were entered using Microsoft Access and analyzed with SAS for Windows Release 9.4 (SAS; Cary, NC, USA). All variables measured at the time of enrollment were considered background or baseline characteristics. The mean and standard deviation or median and range were reported for continuous variables, and frequencies and percentages were used to report categorical data. Continuous measures of the biomarker concentrations were compared among groups using analysis of covariance (ANCOVA; SAS GLM procedure), controlling for the baseline value of the outcome variable. Dichotomous outcomes were defined according to the following cutoffs: (1) low SZC: SZC < 65 µg/dL; (2) anemia: hemoglobin < 11g/dL; (3) iron deficiency: inflammation-adjusted ferritin < 12 µg/L, sTfR > 8.3 mg/L, adjusted body iron stores < 0; (4) iron deficiency anemia: inflammation-adjusted ferritin < 12 µg/L or soluble transferrin receptor > 8.3 mg/L and hemoglobin < 11 g/dL; (5) vitamin A deficiency: retinol-binding protein < 0.81 µmol/L. These outcomes were compared across groups using logistic regression (SAS LOGISTIC procedure), controlling for the baseline value of the respective continuous variable (e.g., hemoglobin for anemia). We also tested for possible effect modification by including a group by effect modifier interaction in the full covariate models. If the *p* value for the interaction term was < 0.05, stratified comparisons were performed to assess the nature of the interaction.

### 2.7. Ethical Considerations

The study was approved by the UCSF Benioff Children’s Hospital Oakland Institutional Review Board, the Colorado Multiple Institutional Review Board, and the Institutional Review Board of the International Center for Diarrheal Disease Research, Bangladesh (icddr,b). The trial was registered at clinicaltrials.gov (accessed on 1 November 2022) (NCT03406793). The research was conducted following the rules of the Declaration of Helsinki.

## 3. Results

**Figure 1:**
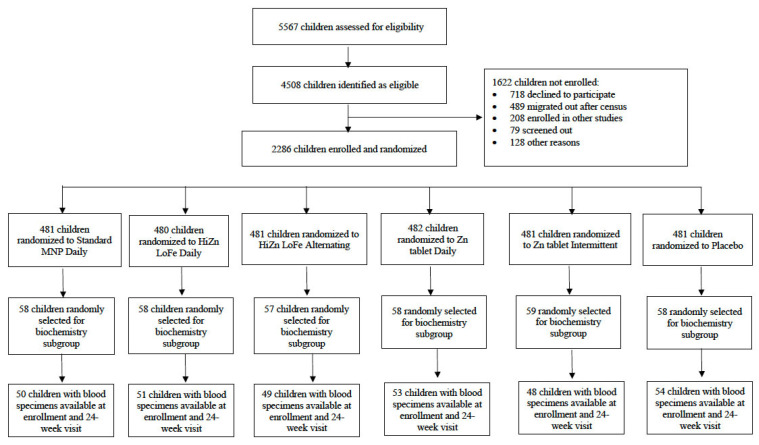
Flowchart of study participants.

As outlined in Figure 1, a total of 5567 children were screened for eligibility and 2886 children were enrolled in the trial. Of these, 348 children were randomly selected for inclusion in the biochemistry subgroup. Blood specimens were available at enrollment and at the end of the 24 week intervention period for 305 (87.6%) of these participants.

Baseline characteristics of the child participants and their families are shown in Table 1. At the time of enrollment, average child age was 9.8 months, and the prevalences of stunting, underweight, and wasting were 25.3%, 16.7%, and 2.6%, respectively. All of the participants’ mothers were married, 94% were housewives, and the average number of years of maternal education was 6.4. Average household monthly income was BDT 17,458 (approximately USD 185) and approximately 20% of households experienced some degree of food insecurity.

As shown in Table 2, micronutrient deficiencies were common at the time of enrollment: 35.5% had low SZC, 60.5% had low serum ferritin concentrations, 66.3% had elevated concentrations of sTfR, and 70.6% of participants were anemic. At the end of the 24 week intervention period, mean SZC varied drastically across groups (*p* < 0.001). After adjusting for baseline values, children in the daily dispersible zinc tablet group exhibited a mean SZC of 92.5 µg/dL, which was significantly higher than all other groups except the HiZn LoFe MNP alternating group (81.3 µg/dL). In contrast, mean 24 week SZC among children in the intermittent zinc tablet group, standard MNP group, and placebo group were 66.7 µg/dL, 71.2 µg/dL, and 73.7 µg/dL, respectively. The corresponding difference in the prevalence of low SZC across groups was similar: at endline, only 5.8% of children in the daily dispersible zinc tablet group had low SZC vs. 35.4% in the intermittent zinc group and 32.7% in the placebo control group. At 18.0%, 12.2%, and 14.2%, the respective prevalence of low SZC was not significantly different among children in the standard MNP, HiZn LoFe daily MNP group, and HiZn LoFe alternating groups.

Regarding iron biomarkers, at 24 weeks, children in the standard MNP, HiZn LoFe MNP daily, and HiZn LoFe alternating groups had significantly higher mean concentrations of inflammation-adjusted ferritin in comparison to children in the daily zinc tablet, intermittent zinc tablet, and placebo control groups. The corresponding prevalences of iron deficiency (defined as an inflammation-adjusted ferritin concentration < 12 µg/L) were 46.0%, 43.1%, and 46.9% in the standard MNP, HiZn LoFe MNP daily, and HiZn LoFe alternating groups vs. 73.8%, 69.5%, and 75.2% in the daily zinc tablet, intermittent zinc tablet, and placebo control groups (Table 2). Likewise, significantly fewer children in the standard MNP and HiZn LoFe daily MNP groups exhibited elevated sTfR concentrations in comparison to children in the zinc tablet daily and placebo powder groups (38.0% and 54.9% vs. 84.9% and 81.8%).

Mean RBP concentration was significantly higher among children in the HiZn LoFe MNP group vs. the intermittent zinc tablet group but otherwise similar across groups.

As hemoglobin concentrations were measured via Hemocue^®^ for all participants in the trial at enrollment and 24 weeks, we had considerably more power to detect statistically significant differences among groups for this endpoint. At 24 weeks, the mean hemoglobin concentration among children in the standard MNP group was higher than any other group. The corresponding prevalence of anemia at this timepoint was comparable among children in the standard MNP and HiZn LoFe MNP daily groups (45.9% and 54.8%, respectively) and significantly lower than the prevalence among children in the zinc tablet daily, intermittent zinc tablet, and placebo powder groups (73.8%, 69.5%, and 75.2%, respectively).

**Figure 2:**
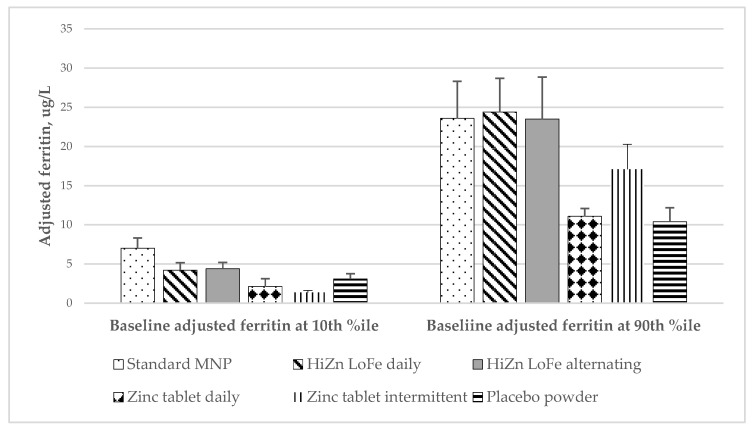
Adjusted ferritin concentrations at 24 weeks shown by adjusted ferritin concentration at baseline.

Our exploratory analyses revealed that baseline status and tertile of adherence significantly modified the effect of the intervention groups on various biomarkers of iron status at 24 weeks (Table 3 and Figure 2). Although our power to detect statistically significant differences between pairs of groups was limited, among children with baseline ferritin concentrations at the tenth percentile, mean ferritin concentrations at 24 weeks tended to be highest in the standard MNP group. However, among children with baseline ferritin concentrations at the 90th percentile, mean ferritin concentrations across the three MNP groups were comparable. Similarly, among children in the highest tertile of consumption, mean ferritin concentrations at 24 weeks were significantly higher among children in the MNP groups in comparison to children receiving dispersible zinc tablets or placebo powder, whereas the differences were less pronounced among children with lower supplement consumption levels. Similar patterns were observed in the responses of other biomarkers of iron status.

## 4. Discussion

In this analysis of iron and zinc biomarker data from a subgroup of young Bangladeshi children who participated in a six-arm, randomized, controlled trial of different zinc supplementation regimens for the prevention of diarrhea and promotion of linear growth, we observed significant differences in the response of SZC and various biomarkers of iron status according to the dose, form, and/or frequency of zinc and iron the child received. Children who received a daily dispersible tablet of zinc alone exhibited an increase in mean SZC that was significantly greater than all other intervention groups except the HiZn LoFe alternating group. In contrast, the response in ferritin was higher among children in the three MNP groups (which contained varying doses of iron) in comparison to children who received the placebo powder or dispersible zinc tablets on a daily or intermittent basis. Group-wise differences in the change in hemoglobin concentration over the course of the 24 week intervention period were generally small. These findings provide a valuable contribution to the existing evidence base regarding the effect of zinc supplementation and MNP interventions on biomarkers of iron and zinc status.

The results of this analysis are particularly relevant within the context of the main ZiPT trial. Though no differences in the incidence or prevalence of diarrhea and minimal differences in linear growth were observed across groups, our ability to detect notable differences in zinc and iron status not only provides support for the high levels of adherence that were reported in the main ZiPT trial but also highlights important nutritional benefits that are conferred by the interventions at a subclinical level. Our study population exhibited remarkably high levels of multiple micronutrient deficiencies at the time of enrollment: more than one-third of participants had low SZC, and the prevalences of low ferritin, elevated sTfR, and anemia were all above 60%. However, by the end of the 24 week intervention period, the prevalence of low SZC had fallen to 5.8% in the dispersible zinc tablet group, and the prevalences of low ferritin concentrations and anemia declined by approximately 20 percentage points in the standard MNP group. These improvements have considerable public health significance given the fact that poor micronutrient status in early childhood has been associated with several adverse health outcomes in the short and long term [5,22,23].

The observed response in SZC is consistent with several studies that have shown that SZC is more responsive when zinc is provided as a stand-alone supplement vs. a point-of-use fortificant added to complementary food [24,25]. In a recent Cochrane analysis of five trials that measured SZC as an outcome, no significant differences in SZC were observed among children who received MNPs vs. no intervention or placebo [14]. Our associated ancillary study that measured changes in the exchangeable zinc pool (EZP) size among ZiPT participants enrolled into the biochemistry subgroups of the HiZn LoFe daily MNP, daily dispersible zinc tablet, and placebo powder arms reported similar results: children in the daily dispersible zinc tablet group exhibited the greatest increase in EZP, whereas the change in EZP was not significantly different between children in the HiZn LoFe daily MNP and placebo powder groups [26]. Likewise, a recent trial of preventive zinc and MNPs in Laos observed that children who received 7 mg of supplemental zinc/day for 32–40 weeks exhibited a significantly greater increase in plasma zinc concentrations in comparison to children who received HiZn LoFe MNPs on a daily basis, therapeutic zinc for treatment of diarrhea, or placebo control [27]. These findings highlight the multifactorial nature of micronutrient deficiencies and the need to acknowledge the potential role of other dietary constituents and non-nutritional factors, such as environmental enteric dysfunction (EED), which may also impact zinc status [28].

Biomarkers of iron status also responded to the various interventions in a way that aligned with our expectations. Although mean ferritin concentrations at endline were significantly higher among children who received MNPs vs. zinc tablets or placebo powders, we did not detect statistically significant differences in ferritin concentrations according to the dose of iron. In the aforementioned LaoZinc trial, children in the HiZn LoFe MNP group similarly exhibited significantly higher ferritin concentrations at endline in comparison to children in the preventive zinc, therapeutic zinc, and control groups. Although children in our trial who received the standard MNP formulation exhibited a mean hemoglobin concentration that was higher than all other study groups, differences were small, and the corresponding prevalence of anemia in the standard MNP group was not significantly lower than the prevalence of anemia among children in the HiZn LoFe daily MNP group. These findings should be carefully considered in relation to the potential adverse effects of MNP formulations containing higher doses of iron, which warrant further research [29]. It is also worth noting that, although considerable improvements in ferritin and hemoglobin concentrations were observed among children receiving iron-containing MNPs, the prevalences of anemia and low ferritin concentrations remained >40% at endline. This raises questions about the bioavailability of iron in MNPs and also highlights the potential effects of “host” factors, such as enteric infections, EED, and inflammation, which can impair iron status and limit the benefits of supplementation and/or home fortification interventions [30,31,32].

Several strengths and limitations of our study deserve mention. Participants were randomly selected from the ZIPT trial, which had an adherence rate of 81.1%. Nearly 90% of participants in this biochemical subgroup provided blood samples at enrollment and endline. Thus, we are confident that our results are generalizable to populations with similar background characteristics. Furthermore, all blood collection, processing, and analysis procedures were conducted by highly trained laboratory staff using trace element-free supplies and followed IZiNCG protocols for the avoidance of zinc contamination. Unfortunately, logistical and budgetary constraints limited the size of the biochemistry subgroup, which likely limited our ability to detect statistically significant differences between groups in some cases. Furthermore, the availability of data on diets and other factors, such as EED, that could influence the absorption of and biological response to the various micronutrients is limited.

To conclude, our trial elucidates the changes in biomarkers of iron and zinc status that occurred when different doses, forms, and frequencies of zinc supplementation were provided to Bangladeshi infants 9–11 months of age for a period of 24 weeks. Children who received MNPs containing 10 mg and 6 mg of iron for the 24 week intervention period exhibited similar improvements in iron status, with no evidence of an adverse interaction with zinc. However, the prevalence of iron deficiency and anemia remained above 40%, highlighting the potential role of non-nutritional “host” factors [13,33]. Although improvements in SZC trended higher among children who received MNPs containing 10 mg of zinc vs. 4.1 mg of zinc in the standard MNP formulation, they were not as large as the increases observed among children who received daily dispersible tablets with 10 mg of zinc alone. These findings underscore the importance of evaluating the doses of micronutrients in supplementation and point-of-use fortification products in relation to the target population’s baseline micronutrient status, usual dietary intake, and complementary feeding patterns and the existence of other possible interventions to improve nutritional status.

## Figures and Tables

**Table 1:** Baseline characteristics of children in the biochemistry subgroup and their mothers by group assignment ^1,2^.

	Standard MNP	HiZn LoFe Daily (*n* = 58)	HiZn LoFe Alternating (*n* = 57)	Zn Tablet Daily (*n* = 58)	Zn Tablet Intermittent (*n* = 59)	Placebo Control (*n* = 58)
***Child characteristics***						
Age, months	9.72 ± 0.82	9.83 ± 0.92	9.76 ± 0.83	9.70 ± 0.93	9.77 ± 0.82	9.86 ± 0.86
Male	29 (50.0)	29 (50.0)	29 (50.9)	29 (50.0)	29 (49.2)	29 (50.0)
Breastfeeding	58 (100)	52 (89.7)	54 (94.7)	54 (93.1)	57 (96.6)	53 (91.4)
Length-for-age *Z*-score	−1.08 ± 1.01	−1.43 ± 1.00	−1.16 ± 1.19	−1.21 ± 1.06	−1.22 ± 1.02	−1.45 ± 1.12
Weight-for-age *Z*-score	−0.73 ± 1.02	−1.39 ± 0.96	−0.89 ± 1.07	−0.91 ± 1.17	−0.96 ± 0.93	−1.29 ± 1.06
Weight-for-length-*Z* score	−0.17 ± 1.00	−0.80 ± 0.88	−0.32 ± 0.85	−0.32 ± 1.06	−0.39 ± 0.86	−0.64 ± 0.92
MUAC, cm	14.1 ± 0.96	13.6 ± 0.83	14.0 ± 0.94	14.1 ± 1.13	14.1 ± 0.90	13.7 ± 0.93
Child healthy at enrollment	14 (24.1)	15 (25.9)	21 (36.8)	11 (19.0)	22 (37.3)	15 (25.9)
***Maternal characteristics***						
Age, years	23.2 ± 5.35	24.8 ± 4.44	25.2 ± 6.17	25.4 ± 5.06	24.9 ± 5.39	24.4 ± 4.96
Currently married	58 (100)	58 (100)	57 (100)	58 (100)	59 (100)	58 (100)
Occupation						
Housewife	55 (94.8)	56 (96.6)	54 (94.7)	55 (94.8)	53 (89.8)	54 (93.1)
Work outside home	2 (3.4)	2 (3.4)	3 (5.3)	2 (3.4)	6 (10.2)	4 (6.9)
Work from home	1 (1.7)	0 (0)	0 (0)	1 (1.7)	0 (0)	0 (0)
Years of education completed	6.83 ± 4.34	5.76 ± 3.81	6.60 ± 4.44	5.62 ± 3.65	7.07 ± 4.01	6.40 ± 3.80
Number of live births in lifetime	1.72 ± 0.83	1.91 ± 0.88	1.88 ± 1.10	2.07 ± 1.06	1.63 ± 0.79	1.86 ± 1.08
***Socioeconomic characteristics***						
Number of people who sleep in the household	4.47 ± 2.50	4.83 ± 1.68	4.58 ± 1.99	4.67 ± 1.77	4.42 ± 1.62	4.72 ± 1.70
Main source of drinking water						
Piped into dwelling	52 (89.7)	1 (1.7)	47 (82.5)	3 (5.2)		3 (5.2)
Piped to yard/plot	4 (6.9)	33 (56.9)	9 (15.8)	33 (56.9)	52 (88.1)	36 (62.1)
Public tap/stand pipe	1 (1.7)	18 (31.0)	0 (0)	19 (32.8)	7 (11.9)	15 (25.9)
Tube well or borehole	1 (1.7)	4 (6.9)	1 (1.8)	3 (5.2)	0 (0)	3 (5.2)
Other	0 (0)	2 (3.4)	0 (0)	0 (0)	0 (0)	1 (1.7)
Flush toilet use	57 (98.3)	50 (86.2)	57 (100)	55 (94.8)	59 (100)	50 (86.2)
Protected toilet use ^3^	7 (12.1)	5 (8.6)	8 (14.0)	4 (6.9)	7 (11.9)	3 (5.2)
Hygiene score ^4^	12.4 ± 2.38	12.9 ± 2.33	12.7 ± 2.29	12.9 ± 2.55	12.7 ± 2.19	12.5 ± 2.42
Asset score ^5^	5.02 ± 2.21	5.48 ± 2.09	5.49 ± 2.77	5.14 ± 2.38	5.59 ± 2.45	5.16 ± 2.38
Average monthly income for the entire household, BDT ^6^	18,078 ± 8443	13,853 ± 8325	22,000 ± 11,720	16,621 ± 12,421	18,864 ± 11,264	15,388 ± 9618
HFIAS classification ^7^						
Food-secure	44 (75.9)	46 (79.3)	46 (80.7)	47 (81.0)	41 (69.5)	53 (91.4)
Mildly food-insecure	3 (5.2)	2 (3.4)	3 (5.3)	2 (3.4)	7 (11.9)	0 (0)
Moderately food-insecure	6 (10.3)	5 (8.6)	1 (1.8)	4 (6.9)	3 (5.1)	3 (5.2)
Severely food-insecure	5 (8.6)	5 (8.6)	7 (12.3)	5 (8.6)	8 (13.6)	2 (3.4)

^1^ Values are means ± SD or *n* (%). ^2^ Standard MNP: standard MNP formulation, consumed daily; HiZn LoFe daily: MNP formulation with 10 mg zinc and 6 mg iron, consumed daily; HiZn LoFe alternating: MNP formulations with 10 mg zinc and 6 mg iron and with 10 mg zinc and no iron consumed on alternating days; Zn tablet daily: 10 mg dispersible zinc tablet, consumed daily; Zn tablet intermittent: 10 mg dispersible zinc tablet, consumed on a daily basis for 14 days at enrollment and after 12 weeks of follow-up, placebo tablet on remaining days; Placebo control: placebo powder, consumed daily. ^3^ A protected toilet was defined as a toilet that flushed to a piped sewer system, septic tank, or pit latrine. ^4^ Hygiene score was calculated as the sum of the scores for the following variables: “Wash hands after helping child defecate”; “Wash hands before preparing food”; “Wash hands after using toilet”; and “Uses toilet paper”. Each variable was scored as follows: 1 = never, 2 = rarely, 3 = sometimes, 4 = always. Asset score could range from 4 to 16. ^5^ Asset scores were the sum of two subscores: asset score 1, which was the sum of the following possessions: iron, chair/bench, sofa, table, computer, fridge, motorcycle, and bank account; asset score 2, which was the sum of the following possessions: electric fan, television, mattress, mobile phone. Asset score could range from 0 to 12. ^6^ USD 1 was equivalent to approximately BDT 85 at the time of the study ^7^ HFIAS: Household Food Insecurity Access Scale.

**Table 2:** Biomarkers of micronutrient status and inflammation at enrollment and at the 24 week visit, by group assignment ^1,2^.

	Standard MNP	HiZn LoFe Daily	HiZn LoFe Alternating	Zn Tablet Daily	Zn Tablet Intermittent	Placebo Control	*p* Value
***Biomarker at enrollment***	*n* = 58	*n* = 58	*n* = 57	*n* = 58	*n* = 59	*n* = 57	
Serum zinc, ug/dL ^3^	70.0 ± 9.97	70.5 ± 10.09	67.8 ± 12.2	66.7 ± 12.8	70.2 ± 12.0	67.8 ± 14.4	0.40
Serum zinc < 65 ug/dL ^3^	15 (25.9)	17 (29.3)	24 (42.9)	28 (48.3)	15 (25.4)	24 (42.1)	0.03
Hemoglobin, g/dL ^4^	10.4 ± 1.16	10.3 ± 1.00	10.3 ± 1.12	10.3 ± 1.04	10.4 ± 1.19	10.3 ± 1.05	0.48
Hemoglobin < 11 g/dL ^4^	334 (69.4)	345 (71.9)	333 (69.2)	357 (74.1)	322 (66.9)	347 (72.1)	0.18
Adjusted ferritin, ug/L ^5^	6.26 ± 7.02 ^b^	10.9 ± 9.91 ^a^	6.05 ± 5.47 ^b^	9.87 ± 9.65 ^a,b^	7.69 ± 9.26 ^a,b^	9.15 ± 9.08 ^a,b^	0.007
Adjusted ferritin < 12 ug/L ^5^	38 (65.5)	29 (50.0)	43 (75.4)	34 (58.6)	35 (59.3)	31 (54.4)	0.10
sTfR, mg/L ^6^	12.2 ± 5.31 ^a,b^	9.71 ± 3.49 ^b^	10.4 ± 4.66 ^a,b^	11.1 ± 5.00 ^a,b^	10.5 ± 4.95 ^a,b^	12.4 ± 6.43 ^a^	0.03
sTfR ^6^ > 8.3 mg/L ^6^	45 (77.6)	38 (65.5)	33 (57.9)	40 (69.0)	33 (55.9)	41 (71.9)	0.12
Adjusted BIS, mg/kg body weight ^6,7^	−3.69 ± 5.15 ^b^	−0.93 ± 3.98 ^a^	−3.24 ± 4.23 ^a,b^	−1.69 ± 4.44 ^a,b^	−2.38 ± 5.54 ^a,b^	−2.33 ± 4.63 ^a,b^	0.02
Adjusted BIS < 0 ^6,7^	43 (74.1)	30 (51.7)	42 (73.7)	34 (58.6)	36 (61.0)	34 (59.6)	0.09
Iron deficiency anemia	34 (58.6)	25 (43.1)	30 (52.6)	32 (55.2)	26 (44.1)	24 (42.1)	0.33
RBP, umol/L ^6^	1.08 ± 0.22 ^a,b^	0.98 ± 0.23 ^b^	1.14 ± 0.23 ^a^	0.96 ± 0.30 ^b^	1.12 ± 0.25 ^a^	0.95 ± 0.28 ^b^	<0.0001
RBP < 0.81 umol/L ^6^	4 (6.9) ^a,b^	10 (17.2) ^a,b^	2 (3.5) ^a^	14 (24.1) ^a,b^	4 (6.8) ^a,b^	16 (28.1) ^b^	0.001
CRP, mg/L ^6^	0.23 ± 0.46	0.23 ± 0.41	0.22 ± 0.38	0.32 ± 0.63	0.14 ± 0.25	0.21 ± 0.43	0.31
CRP> 5 mg/L ^6^	5 (8.6)	4 (6.9)	2 (3.5)	5 (8.6)	2 (3.4)	6 (10.5)	0.61
AGP, g/L ^6^	0.68 ± 0.30	0.62 ± 0.33	0.66 ± 0.30	0.67 ± 0.38	0.614 ± 0.27	0.604 ± 0.33	0.66
AGP > 1 g/L ^6^	9 (15.5)	13 (22.4)	11 (19.3)	14 (24.1)	7 (11.9)	9 (15.8)	0.53
***Biomarker at 24 week visit***	*n* = 50	*n* = 51	*n* = 49	*n* = 53	*n* = 48	*n* = 54	
Serum zinc, ug/dL ^7^	71.2 ± 2.38 ^c,d^	80.7 ± 2.56 ^b,c^	81.3 ± 2.60 ^a,b^	92.5 ± 2.86 ^a^	66.7 ± 2.28 ^d^	73.7 ± 2.22 ^b–d^	<0.0001
Serum zinc < 65 ug/dL	9 (18.0) ^a,b^	6 (12.2) ^a,b^	7 (14.3) ^a,b^	3 (5.8) ^a^	17 (35.4) ^b^	18 (32.7) ^b^	0.0003
Hemoglobin, g/dL ^3^	11.0 ± 0.05 ^a^	10.7 ± 0.05 ^b^	10.7 ± 0.05 ^b^	10.2 ± 0.05 ^c^	10.21 ± 0.0 ^c^	10.2 ± 0.05 ^c^	<0.0001
Hemoglobin < 11 g/dL	203 (45.9) ^a^	247 (54.8) ^a,b^	264 (60.4) ^b^	324 (73.8) ^c^	303 (69.5) ^c^	340 (75.2) ^c^	<0.0001
Adjusted ferritin, ug/L ^4^	14.4 ± 2.01 ^a^	10.8 ± 1.42 ^a^	10.9 ± 1.44 ^a^	5.10 ± 0.65 ^b^	5.09 ± 0.73 ^b^	5.68 ± 0.72 ^b^	<0.0001
Adjusted ferritin < 12 ug/L	23 (46.0) ^a^	22 (43.1) ^a^	23 (46.9) ^a,b^	42 (79.2) ^c^	36 (75.0) ^b,c^	46 (83.6) ^c^	<0.0001
sTfR^6^, mg/L	7.72 ± 0.2 ^d^	10.0 ± 0.56 ^b–d^	9.95 ± 0.56 ^c,d^	12.9 ± 0.62 ^a^	12.3 ± 0.68 ^a,b^	12.2 ± 0.60 ^a–c^	<0.0001
sTfR^6^ > 8.3 mg/L	19 (38.0) ^a^	28 (54.9) ^a^	31 (63.3) ^a–c^	45 (84.9) ^c^	28 (58.3) ^a,b^	45 (81.8) ^b,c^	<0.0001
Adjusted BIS, mg/kg body weight ^6,7^	0.78 ± 0.60 ^a^	−0.95 ± 0.56 ^a^	−0.87 ± 0.57 ^a^	−4.73 ± 0.55 ^b^	−4.40 ± 0.61 ^b^	−4.04 ± 0.54 ^b^	<0.0001
Adjusted BIS < 0 mg/kg body weight ^6,7^	20 (40.0) ^a^	24 (47.1) ^a^	27 (55.1) ^a^	44 (83.0) ^b^	33 (68.8) ^a,b^	47 (85.5) ^b^	<0.0001
Iron deficiency anemia	14 (28.0) ^a^	17 (33.3) ^a,b^	15 (30.6) ^a–c^	32 (61.5) ^a–c^	26 (54.2) ^b,c^	39 (70.9) ^c^	<0.0001
RBP, umol/L ^6^	0.93 ± 0.04 ^a,b^	1.09 ± 0.04 ^a^	0.91 ± 0.04 ^a,b^	0.97 ± 0.04 ^a,b^	0.84 ± 0.04 ^b^	1.02 ± 0.04 ^a,b^	0.008
RBP < 0.81 umol/L ^6^	8 (16.0)	4 (7.8)	8 (16.3)	8 (15.1)	12 (25.0)	6 (10.9)	0.20
CRP, mg/L ^6^	0.37 ± 0.15	0.17 ± 0.06	0.60 ± 0.23	0.17 ± 0.06	0.46 ± 0.19	0.32 ± 0.12	0.28
CRP > 5 mg/L ^6^	6 (12.0)	7 (13.7)	8 (16.3)	8 (15.1)	9 (18.8)	10 (18.2)	0.94
AGP, g/L ^6^	0.60 ± 0.06 ^b^	0.69 ± 0.06 ^a,b^	0.83 ± 0.07 ^a^	0.64 ± 0.05 ^a,b^	0.67 ± 0.06 ^a,b^	0.76 ± 0.06 ^a,b^	0.03
AGP > 1 g/L ^6^	10 (20.0) ^a,b^	11 (21.6) ^a,b^	20 (40.8) ^b^	7 (13.2) ^a^	15 (31.3) ^a,b^	13 (23.6) ^a,b^	0.04

^1^ Values are means ± SD or *n* (%). Labeled values in a row without a common superscript letter differ, *p* < 0.05 (Tukey–Kramer test). ^2^ Standard MNP: standard MNP formulation, consumed daily; HiZn LoFe daily: MNP formulation with 10 mg zinc and 6 mg iron, consumed daily; HiZn LoFe alternating: MNP formulations with 10 mg zinc and 6 mg iron and with 10 mg zinc and no iron consumed on alternating days; Zn tablet daily: 10 mg dispersible zinc tablet, consumed daily; Zn tablet intermittent: 10 mg dispersible zinc tablet, consumed on a daily basis for 14 days at enrollment and after 12 weeks of follow-up, placebo tablet on remaining days; Placebo control: placebo powder, consumed daily. ^3^ At enrollment, serum zinc concentrations were available for 56 children in the HiZn LoFe alternating group and 56 children in the placebo control group. At the 24 week visit, serum zinc concentrations were available for 49 children in the HiZn LoFe daily group, 48 children in the HiZn LoFe alternating group, and 52 children in the zinc tablet daily group. All other reported Ns apply to the serum zinc data. ^4^ At enrollment, hemoglobin concentration data were available for 481 children in the standard MNP group, 480 children in the HiZn LoFe daily group, 482 children in the HiZn LoFe alternating group, 482 children in the zinc tablet daily group, 481 children in the zinc tablet intermittent group, and 481 children in the placebo control group. At the 24 week visit, hemoglobin concentration data were available for 442 children in the standard MNP group, 451 children in the HiZn LoFe daily group, 437 children in the HiZn LoFe alternating group, 439 children in the zinc tablet daily group, 436 children in the zinc tablet intermittent group, and 452 children in the placebo control group. ^5^ Ferritin concentration was adjusted for CRP and AGP using the BRINDA regression correction approach. ^6^ sTfR: soluble transferrin receptor; BIS: body iron stores; RBP: retinol-binding protein; CRP: C-reactive protein; AGP: alpha-1-acid glycoprotein. ^7^ Body iron stores = −(log10(sTfR/(ferritin/1000)) −2.8229)/0.1207. In this formula, soluble transferrin receptor and ferritin were adjusted for CRP and AGP using the BRINDA regression correction approach.

**Table 3:** Effect modification analysis for iron biomarker concentrations at 24 weeks ^1,2^.

Variable	Effect Modifier (X)	Standard MNP	HiZn LoFe Daily	HiZn LoFe Alternating	Zn Tablet Daily	Zn Tablet Intermittent	Placebo Control	Group by X *p*-Value	Group within X *p*-Value
Adj. ferritin at 24 weeks, ug/L ^3^	Baseline adj. ferritin at 10th %ile ^3^	7.04 ± 1.29 ^a^	4.22 ± 0.96 ^a,b^	4.39 ± 0.81 ^a,b^	2.14 ± 0.48 ^b,c^	1.37 ± 0.25 ^c^	3.10 ± 0.66 ^a–c^	0.004	<0.0001
	Baseline adj. ferritin at 90th %ile ^3^	23.6 ± 4.71 ^a,b^	24.4 ± 4.31 ^a^	23.5 ± 5.36 ^a–c^	11.1 ± 1.84 ^b,c^	17.1 ± 3.19 ^a–c^	10.4 ± 1.78 ^c^		0.0002
	Lowest tertile of consumption	7.61 ± 1.90	6.66 ± 1.61	9.56 ± 2.0	5.63 ± 1.48	6.27 ± 1.68	5.79 ± 1.31	0.01	0.62
	Mid tertile of consumption	11.8 ± 2.61 ^a^	12.02 ± 2.08 ^a^	10.2 ± 2.06 ^a^	5.51 ± 0.93 ^a^	5.48 ± 1.04 ^a^	6.48 ± 1.17 ^a^		0.0003
	Highest tertile of consumption	22.0 ± 3.89 ^a^	12.3 ± 2.44 ^a^	12.8 ± 2.55 ^a^	4.19 ± 0.83 ^b^	4.24 ± 0.85 ^b^	4.85 ± 0.89 ^b^		<0.0001
Adj. ferritin < 12 ug/L at 24 weeks ^3^	Baseline adj. ferritin at 10th %ile ^3^	63.5 ± 10.3 ^a^	88.9 ± 7.75 ^a^	83.7 ± 8.64 ^a,b^	99.9 ± 0.15 ^b^	98.8 ± 1.65 ^a,b^	98.9 ± 1.56 ^a,b^	0.03	0.004
	Baseline adj. ferritin at 90th %ile ^3^	25.9 ± 10.0 ^a,b^	13.6 ± 7.01 ^a^	5.73 ± 4.92 ^a^	46.0 ± 13.4 ^a,b^	37.4 ± 14.5 ^a,b^	64.3 ± 11.02 ^b^		0.001
sTfR at 24 weeks, mg/L ^3,4^	Baseline sTfR at 10th %ile ^3^	6.99 ± 0.65 ^a,b^	6.86 ± 0.59 ^b^	7.19 ± 0.63 ^a,b^	9.39 ± 0.68 ^a^	7.83 ± 0.63 ^a,b^	9.28 ± 0.68 ^a^	<0.0001	0.007
	Baseline sTfR at 90th %ile ^3^	10.6 ± 0.87 ^c^	15.3 ± 1.47 ^b,c^	15.1 ± 1.14 ^b,c^	19.2 ± 1.37 ^a,b^	23.2 ± 1.52 ^a^	17.3 ± 0.98 ^b^		<0.0001
BIS (mg/kg body weight) ^4,5^	Baseline body iron at 10th %ile	−1.81 ± 0.88 ^a^	−5.41 ± 1.15 ^a–c^	−4.44 ± 0.91 ^a,b^	−8.98 ± 1.08 ^c,d^	−10.9 ± 0.89 ^d^	−7.23 ± 0.96 ^b–d^	0.0002	<0.0001
	Baseline body iron at 90th %ile	3.48 ± 0.89 ^a,b^	4.07 ± 0.81 ^a^	3.83 ± 0.97 ^a,b^	−0.15 ± 0.74 ^c^	2.62 ± 0.83 ^a–c^	0.75 ± 0.82 ^b,c^		0.0002
	Lowest tertile of consumption	−0.84 ± 1.15	−2.24 ± 1.12	0.21 ± 0.98	−2.51 ± 1.21	−2.75 ± 1.24	−2.46 ± 1.05	0.007	0.32
	Mid tertile of consumption	1.02 ± 1.02 ^a^	0.29 ± 0.80 ^a^	0.49 ± 0.93 ^a^	−3.46 ± 0.78 ^b^	−2.47 ± 0.87 ^a,b^	−2.62 ± 0.83 ^a,b^		<0.0001
	Highest tertile of consumption	3.20 ± 0.81 ^a^	0.89 ± 0.91 ^a^	0.23 ± 0.92 ^a,b^	−5.90 ± 0.91 ^c^	−4.70 ± 0.93 ^c^	−3.06 ± 0.84 ^b,c^		<0.0001
BIS < 0 ^4,5^	Baseline BIS at 10th %ile	42.9 ± 11.2 ^a^	88.9 ± 7.92 ^a,b^	85.2 ± 9.14 ^a,b^	99.8 ± 0.37 ^b^	99.6 ± 0.83 ^b^	97.4 ± 2.77 ^a,b^	0.0291	0.0004
	Baseline body iron at 90th %ile	17.5 ± 8.25 ^a,b^	12.0 ± 6.33 ^a^	6.50 ± 5.04 ^a^	46.3 ± 11.6 ^b^	15.3 ± 9.40 ^a,b^	42.8 ± 11.9 ^a,b^		0.01

^1^ Values are means ± SD or *n* (%). Labeled values in a row without a common superscript letter differ, *p* < 0.05 (Tukey–Kramer test). ^2^ Standard MNP: standard MNP formulation, consumed daily; HiZn LoFe daily: MNP formulation with 10 mg zinc and 6 mg iron, consumed daily; HiZn LoFe alternating: MNP formulations with 10 mg zinc and 6 mg iron and with 10 mg zinc and no iron consumed on alternating days; Zn tablet daily: 10 mg dispersible zinc tablet, consumed daily; Zn tablet intermittent: 10 mg dispersible zinc tablet, consumed on a daily basis for 14 days at enrollment and after 12 weeks of follow-up, placebo tablet on remaining days; Placebo control: placebo powder, consumed daily. ^3^ Ferritin and sTfR were adjusted for CRP and AGP using the BRINDA regression correction approach. ^4^ sTfR: soluble transferrin receptor; BIS: body iron stores; CRP: C-reactive protein; AGP: alpha-1-acid glycoprotein. ^5^ Body iron stores = −(log10(sTfR/(ferritin/1000)) − 2.8229)/0.1207. In this formula, soluble transferrin receptor and ferritin were adjusted for CRP and AGP using the BRINDA regression correction approach.

## Data Availability

Data described in the manuscript, code book, and analytic code will be made available upon request pending application and approval.

## Supplementary Materials

The following supporting information can be downloaded at: https://www.mdpi.com/article/10.3390/nu14245334/s1, Table S1: Characteristics of the study interventions.
