# Supplementary material for: Effects of Different Doses, Forms, and Frequencies of Zinc Supplementation on Biomarkers of Iron and Zinc Status among Young Children in Dhaka, Bangladesh
Source: Nutrients. 2022 Dec 15;14(24):5334. doi: 10.3390/nu14245334 (PMC9781687; doi:10.3390/nu14245334)
Supplement: Supplementary file 1 [file nutrients-14-05334-s001.zip › nutrients-2039715-supplementary.pdf]

Table S1. Characteristics of the study interventions

Study Group	Form	Micronutrient Content	Frequency of consumption
Standard MNP	Powder	Vitain A (as Vitamin A acetate): 400 µg<br>Vitamin D (as Cholecalciferol): 5 µg<br>Vitamin E (as dl-alpha-tocopheryl acetate): 5 mg<br>Vitamin C (as Ascorbic acid): 30 mg<br>Thiamine (as Thiamin mononitrate): 0.5 mg<br>Riboflavin: 0.5 mg<br>Niacin (as Niacinamide): 6 mg<br>Vitamin B6 (as Pyridoxine hydrochloride): 0.5 mg<br>Vitamin B12 (as Cyanocobalamin): 0.9 mg<br>Folic acid: 150 µg<br>Iron (as Ferrous fumarate): 10 mg<br>Zinc (as Zinc gluconate): 4.1 mg<br>Copper (as Copper sulfate, anhydrous): 0.56 mg<br>Selenium (as Sodium selenite): 17 µg<br>Iodine (as Potassium iodate): 90 µg	Daily for 24 weeks
HiZn LoFe Daily	Powder	Same as Standard MNP, except with 10 mg zinc and 6 mg iron	Daily for 24 weeks
HiZn LoFe Alternating	Powder	Same as Standard MNP except with 10 mg zinc, and 6 mg iron and no iron on alternating days	Daily for 24 weeks
Zn Tablet Daily	Dispersible tablet	Zinc (as Zinc Sulfate Monohydrate): 10 mg	Daily for 24 weeks
Zn Tablet Intermittent	Dispersible tablet	Zinc: (as Zinc Sulfate Monohydrate): 10 mg	Daily for 14 days at baseline and 12 weeks, placebo tablet on all other days
Placebo Control	Powder	None	Daily for 24 weeks
